# Prediction models of incontinence and sexual function one year after radical prostatectomy based on data from 20 164 prostate cancer patients

**DOI:** 10.1371/journal.pone.0295179

**Published:** 2023-12-01

**Authors:** Nora Tabea Sibert, Tobias Kurth, Clara Breidenbach, Simone Wesselmann, Günther Feick, Ernst-Günter Carl, Sebastian Dieng, Mohamad Hatem Albarghouth, Atiqullah Aziz, Stefan Baltes, Elisabeth Bartolf, Jens Bedke, Andreas Blana, Marko Brock, Stefan Conrad, Christopher Darr, Florian Distler, Konstantinos Drosos, Gregor Duwe, Amr Gaber, Markus Giessing, Nina Natascha Harke, Axel Heidenreich, Sameh Hijazi, Andreas Hinkel, Björn Theodor Kaftan, Shatlyk Kheiderov, Thomas Knoll, Gerd Lümmen, Inga Peters, Bülent Polat, Valentin Schrodi, Jens-Uwe Stolzenburg, Zoltan Varga, Julius von Süßkind-Schwendi, Vahudin Zugor, Christoph Kowalski

**Affiliations:** 1 German Cancer Society (DKG), Berlin, Germany; 2 Institute of Public Health, Charité-Universitätsmedizin Berlin, Berlin, Germany; 3 Bundesverband Prostatakrebs Selbsthilfe, Bonn, Germany; 4 OnkoZert GmbH, Neu-Ulm, Germany; 5 Sankt Georg Hospital, Leipzig, Germany; 6 München Klinik Bogenhausen, München, Germany; 7 KRH Klinikum Region Hannover, Klinikum Siloah—Oststadt—Heidehaus, Hannover, Germany; 8 Ingolstadt Hospital, Ingolstadt, Germany; 9 University Hospital Tübingen, Tübingen, Germany; 10 Klinikum Fürth, Fürth, Germany; 11 Ruhr-University Bochum, Marien Hospital, Herne, Germany; 12 DIAKOVERE Friederikenstift, Hannover, Germany; 13 University Hospital Essen, Essen, Germany; 14 Klinikum Nürnberg, Nürnberg, Germany; 15 University Hospital Jena, Jena, Germany; 16 University Hospital Mainz, Mainz, Germany; 17 Carl-Thiem-Klinikum, Cottbus, Germany; 18 Kliniken Maria Hilf, Mönchengladbach, Germany; 19 Medizinische Hochschule Hannover Hospital, Hannover, Germany; 20 University Hospital Köln, Cologne, Germany; 21 Klinikum Ibbenbüren, Ibbenbüren, Germany; 22 Franziskus Hospital Bielefeld, Bielefeld, Germany; 23 Klinikum Lüneburg, Lüneburg, Germany; 24 Johanniter Krankenhaus Oberhausen, Oberhausen, Germany; 25 Klinikum Sindelfingen-Böblingen, Sindelfingen, Germany; 26 GFO Kliniken Troisdorf, Troisdorf, Germany; 27 Krankenhaus Nordwest, Frankfurt am Main, Germany; 28 University Hospital Würzburg, Würzburg, Germany; 29 Academic Hospital Braunschweig, Braunschweig, Germany; 30 University Clinic Leipzig, Leipzig, Germany; 31 SRH Kliniken Landkreis Sigmaringen, Sigmaringen, Germany; 32 Schwarzwald-Baar Klinikum, Villingen-Schwenningen, Germany; 33 Hospital Sozialstiftung Bamberg, Bamberg, Germany; Justus Liebig University Giessen, GERMANY

## Abstract

**Background:**

Incontinence and sexual dysfunction are long-lasting side effects after surgical treatment (radical prostatectomy, RP) of prostate cancer (PC). For an informed treatment decision, physicians and patients should discuss expected impairments. Therefore, this paper firstly aims to develop and validate prognostic models that predict incontinence and sexual function of PC patients one year after RP and secondly to provide an online decision making tool.

**Methods:**

Observational cohorts of PC patients treated between July 2016 and March 2021 in Germany were used. Models to predict functional outcomes one year after RP measured by the EPIC-26 questionnaire were developed using lasso regression, 80–20 splitting of the data set and 10-fold cross validation. To assess performance, R^2^, RMSE, analysis of residuals and calibration-in-the-large were applied. Final models were externally temporally validated. Additionally, percentages of functional impairment (pad use for incontinence and firmness of erection for sexual score) per score decile were calculated to be used together with the prediction models.

**Results:**

For model development and internal as well as external validation, samples of 11 355 and 8 809 patients were analysed. Results from the internal validation (incontinence: *R*^2^ = 0.12, *RMSE* = 25.40, sexual function: *R*^2^ = 0.23, *RMSE* = 21.44) were comparable with those of the external validation. Residual analysis and calibration-in-the-large showed good results. The prediction tool is freely accessible: https://nora-tabea.shinyapps.io/EPIC-26-Prediction/.

**Conclusion:**

The final models showed appropriate predictive properties and can be used together with the calculated risks for specific functional impairments. Main strengths are the large study sample (> 20 000) and the inclusion of an external validation. The models incorporate meaningful and clinically available predictors ensuring an easy implementation. All predictions are displayed together with risks of frequent impairments such as pad use or erectile dysfunction such that the developed online tool provides a detailed and informative overview for clinicians as well as patients.

## Introduction

Although being an important treatment strategy with high rates of disease-free survival [1], radical prostatectomy (RP) is associated with possible severe functional impairments [2]. In particular, erectile dysfunction and urinary incontinence as a result of RP should be mentioned here, which are still marked after 24 months and can persist for life in many affected men [3,4]. Treatment providers and patients have addressed this problem for a long time, and studies comparing different modes of RP are now not only examining PC-free survival but also functional outcomes, including incontinence and sexual dysfunction [5]. The preservation of continence and sexual function are essential goals of medical guidelines for treating PC [6,7].

To enable informed and shared decision-making, patients and treating physicians need to consider the expected functional impairments when deciding on a treatment option. One potential tool for measuring functional outcomes is the EPIC-26 (26-item Expanded Prostate Cancer Index Composite). This well-established patient-reported outcome questionnaire is part of the International Consortium on Health Outcomes Measurement (ICHOM) standard data set [8]. The EPIC-26 measures the following five domains (leading to these scores): urinary incontinence, irritative/obstructive symptoms, bowel function, sexual function and vitality/hormonal function [9,10] with especially incontinence and sexual function being negatively affected in patients treated with RP [11]. Further research could underscore, that for incontinence, in particular pad use, and for sexual function, the firmness of erections are most important [12]. This is in accordance with the well-known “trifecta model”, often used for judging quality of RP care [13].

Laviana et al. proposed a prediction tool in 2020 to offer clinicians and patients concise and clinically relevant information about expected outcomes. Yet, this model has not been externally validated. It also does not take surgical approaches or information about patients’ comorbidities into account [14].

Hence, the aim of this paper is to develop a web-based tool for clinicians and patients for incontinence and sexual function one year after RP that extends existing tools by adding surgical approaches and comorbidities. For that the aim is firstly to develop and validate prognostic models that predict incontinence and sexual function based on a continuous scale one year after RP, including information on different surgical approaches (open, robotic or laparoscopic). To inform potential users of those predictions even more, as a second step risk for the clinically relevant symptoms pad use and firmness of erection are calculated dependent on outcome scores and reported together with the predictions.

## Patients and methods

Prognostic prediction models were developed and validated internally and externally, following the TRIPOD framework ([15], cf. S5 File).

### Study population and data sources

Since 2016, the Prostate Cancer Outcomes (PCO) study [16]–initiated by the Movember Foundation–has been conducted within PC centres certified in accordance with the requirements of the German Cancer Society (DKG) [17] in order to record functional outcomes after PC treatment. The PCO study is part of the TrueNTH Global Registry, which currently involves healthcare providers from 15 countries around the world [18] and collects data based on the ICHOM standard data set for localised PC [19]. PCO is on-going and more than 45 000 enrolled patients up until now [16]. For this article, only patients who received a RP were included. Latest data access for this research purpose was 30^th^ May 2022.

### Outcome definition, predictors set and handling of missing data

This study investigates on the two most common functional outcomes after RP—incontinence and sexual impairment–measured by the EPIC-26 questionnaire [9] on continuous scales (0–100) one year after RP. The time of prediction should be before treatment (decision), and besides two overall prediction models for incontinence and sexual function, models for different surgical approaches (open, robotic, laparoscopic) are presented (cf. S1 File).

Candidate predictors were chosen literature-based, including clinical (i.e. PSA, Gleason, cT, cN, comorbidities) and sociodemographic (i.e. age, insurance status) information, baseline EPIC-26 scores (before RP), as well as surgical approach (robot-assisted, open, laparoscopic). Besides the sociodemographic information, all information was obtained using routinely documented certification data of the participating centres and thus missingness rates were low as already described elsewhere [20].

K-nearest neighbour imputation [21] was used for handling missing data (with k = 6) using the R package VIM [22]. Additional missing value analysis might help to elucidate on the impact of missingness (cf. S3 File).

For the second aim of this study, incontinence and sexual function scores one year after RP were categorised into deciles and the risks for the outcomes “pad use” (item 3 of the EPIC-26) and “erection not firm enough for intercourse, foreplay or masturbation” (item 9 –dichotomisation following the NIH Consensus Development Panel of Impotence’s definition of erectile dysfunction [23]) were calculated for each score’s decile (for an enhanced readability of the paper, the item “erection not firm enough for intercourse, foreplay or masturbation” is referred to as “erectile dysfunction” from now on, although the authors acknowledge that erectile dysfunction can incorporate more impairments).

### Statistical analysis

Descriptive statistics are presented stratified by surgical approach (categories: open, robotic, laparoscopic or not specified). For the second aim of this study, the risk for patients of either pad use or erection dysfunction one year after RP was calculated stratified by the corresponding EPIC-26 score for further online presentation using the application. For model development and selection of suitable predictors, Tibishirani’s least absolute shrinkage and selection operator (lasso) regression (for linear outcome) was applied [24] using the R packages *caret* and *glmnet* [25,26]. To avoid over-fitting, a 10-fold cross-validation was performed to find the best *λ* fit within the lasso.

For assessing the model performance, the available data set was split randomly into a training and testing data set (80–20 split), with only the training data set being used for model development. Established parameters (*R*^2^ and root-mean-square error (RMSE)) were calculated by applying the testing data set on the final models (including the intercepts) developed on the training data set to evaluate model performance. Moreover, residuals for the testing data set were analysed, including a graphical display of the results (QQ plots and fitted values vs. residuals). Calibration-in-the-large was assessed by comparing the mean observed to the mean expected scores. Variable importance was assessed using the varImp function of the *caret* package based on the absolute value of the t-test of the shrinked coefficients and scaled between 0 and 100. Results for variable importance are shown in S4 File.

In order to further examine the validity of a given prediction model, an external temporal validation was performed using all follow-up questionnaires after June 2021 (which were not used for model development). By applying the prognostic prediction models to the new data set, *R*^2^ and RMSE were also calculated and compared to the results of the internal validation, and residual and calibration-in-the-large analyses were performed as well.

All model development and validation steps were consecutively performed for data sets stratified by surgical approach (open, robotic or laparoscopic), resulting in six additional prediction models with a special emphasis on the specific RP method (results: cf. S1 File).

The proposed web-based application was coded using the R package *shiny* [27]. For all analysis, R version 4.2.2 was used. Since this study focuses on prediction, following the TRIPOD guideline for reporting prediction model development and validation [15] and state-of-the-art epidemiological predictive methodology [28,29], no confidence intervals or p-values for predictors’ regression coefficients are presented, nor are any statements about the statistical significance of the coefficients made (cf. discussion section, as well).

### Ethics

All analyses performed in this study involving human participants are in accordance with the ethical standards of Ethics Committee of the Medical Association of Berlin and with the 1964 Helsinki declaration and its later amendments or comparable ethical standards. The study protocol of the PCO study was approved by the Ethics Committee of the Medical Association of Berlin (Eth-12/16) which can be contacted via ek@aekb.de. The study was registered in the German Clinical Trials Register (ID: DRKS00010774).

## Results

### Study population

**Fig 1:**
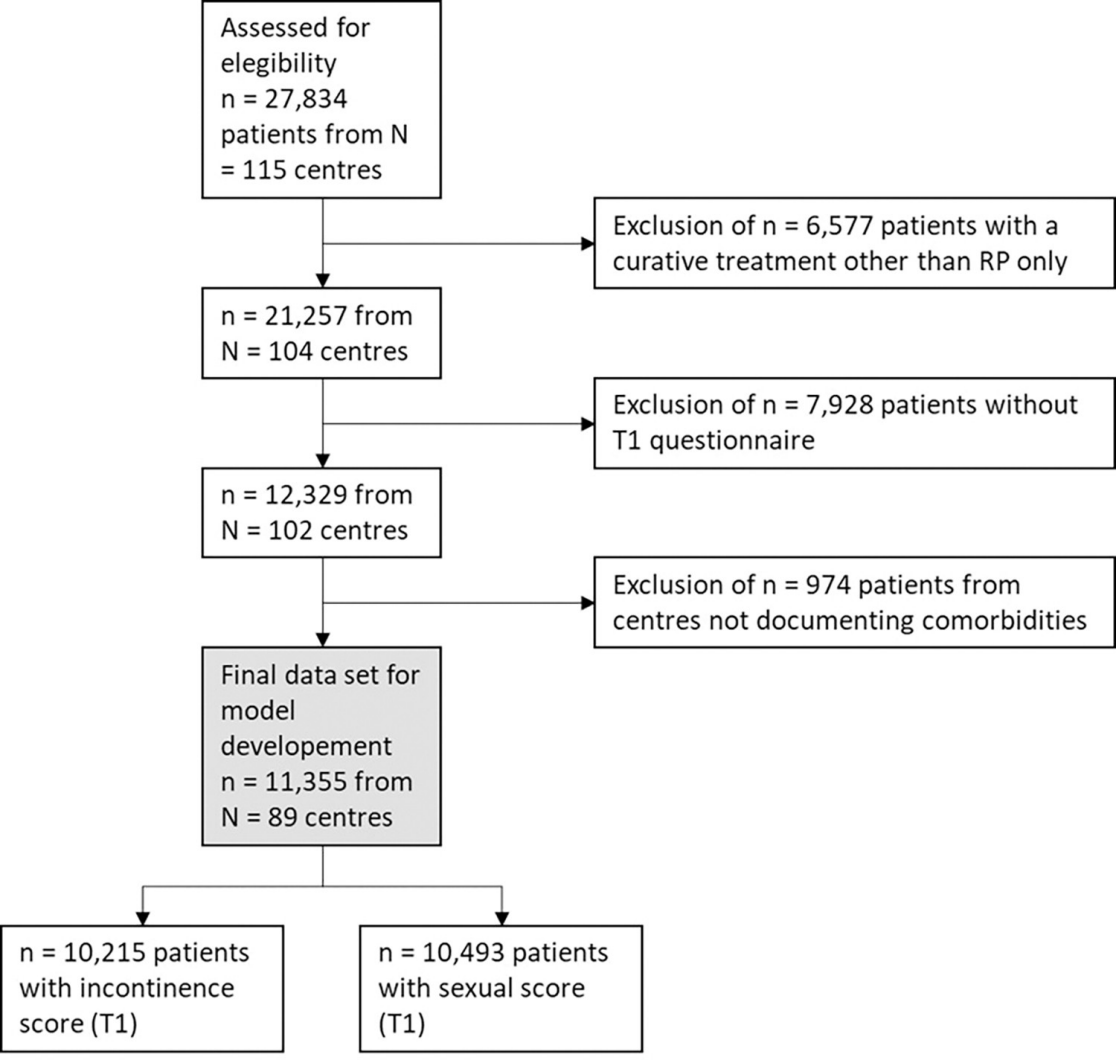
Flow chart for the sample used for model development and internal validation (all T1 questionnaires before June 2021).

For model development and internal validation, a total of 11 355 RP-treated patients with a one-year follow-up questionnaire (T1) and from a centre that documented comorbidities (cf. Patients and Methods) were included (for further information, cf. Fig 1). The basis for this sample was data from the PCO study up to a T1 questionnaire before June 2021. For temporal external validation, an additional sample of 8 809 patients with a T1 questionnaire between June 2021 and May 2022 was used (except for the timing of the T1 questionnaire, the same inclusion criteria were used).

Both cohorts were combined for calculating the risk for pad use and erectile dysfunction resulting in a cohort of 17 473 eligible patients with information on those items.

Median age of patients included in the model development data set was 66 years, and the majority of patients were treated with a robot-assisted RP (5 226 of 11 355 patients, 46.0%) followed by open RP (3 939 of 11 355 patients, 34.7%). For an overview of patients’ characteristics, compare Table 1.

**Table 1:** Patients’ characteristics for 11,355 patients treated by RPE, stratified by surgical approach.

	robotic, N = 5,226^1^	open, N = 3,939^1^	laparoscopic, N = 1,045^1^	not specified, N = 1,145^1^
**Age**	65 (60, 70)	67 (62, 71)	67 (61, 71)	67 (61, 71)
**Insurance**				
Statutory	3,446 (68.1%)	2,631 (68.6%)	797 (78.0%)	869 (77.5%)
Private	1,577 (31.2%)	1,185 (30.9%)	223 (22.1%)	248 (21.8%)
None or other	34 (0.7%)	19 (0.5%)	2 (0.2%)	4 (0.4%)
Unknown	169	104	23	24
**Citizenship**				
German	4,885 (96.6%)	3,759 (98.0%)	1,000 (96.7%)	1,079 (97.3%)
Other	171 (3.4%)	75 (2.0%)	28 (2.7%)	37 (3.3%)
Unknown	170	105	17	29
**Education** ^**2**^				
Lower	2,644 (52.3%)	2,158 (56.3%)	667 (65.3%)	699 (62.7%)
Higher	2,311 (45.7%)	1,614 (42.1%)	340 (33.3%)	395 (35.5%)
Other	78 (1.5%)	51 (1.3%)	13 (1.3%)	14 (1.3%)
None	21 (0.4%)	13 (0.3%)	2 (0.2%)	6 (0.5%)
Unknown	172	103	23	31
**AS (before RP)**	84 (1.6%)	85 (2.2%)	39 (3.7%)	23 (2.0%)
**ADT (before RP)**	53 (1.0%)	55 (1.4%)	6 (0.6%)	7 (0.6%)
**WW (before RP)**	3 (<0.1%)	3 (<0.1%)	1 (<0.1%)	1 (<0.1%)
**PSA level (at diagnosis)**	7 (5, 11)	8 (5, 12)	7 (5, 10)	8 (6, 12)
Unknown	1	0	0	0
**cT (before RP)**				
T0	0 (0%)	2 (<0.1%)	0 (0%)	0 (0%)
T1	3,919 (75.0%)	2,720 (69.1%)	587 (56.2%)	755 (65.9%)
T2	1,226 (23.5%)	1,083 (27.5%)	423 (40.5%)	358 (31.3%)
T3	79 (1.5%)	131 (3.3%)	35 (3.3%)	31 (2.7%)
T4	2 (<0.1%)	3 (<0.1%)	0 (0%)	1 (<0.1%)
**cN (before RP)**				
N0	5,201 (99.5%)	3,902 (99.1%)	1,038 (99.3%)	1,132 (98.9%)
N1	25 (0.5%)	37 (0.9%)	7 (0.7%)	13 (1.1%)
**Gleason score (before RP)** ^**3**^				
Grade 1	1,174 (22.5%)	886 (22.5%)	274 (26.2%)	310 (27.1%)
Grade 2	2,045 (39.1%)	1,471 (37.3%)	400 (38.3%)	442 (38.6%)
Grade 3	1,088 (20.8%)	708 (18.0%)	216 (20.7%)	191 (16.7%)
Grade 4	628 (12.0%)	570 (14.5%)	111 (10.6%)	125 (10.9%)
Grade 5	291 (5.6%)	304 (7.7%)	44 (4.2%)	77 (6.7%)
**Incontinence (EPIC-26)**				
T0	93.7 (12.7)	92.9 (13.6)	93.2 (13.0)	92.1 (14.3)
T1	75 (27.0)	72.7 (27.4)	71.3 (26.4)	72.7 (28.2)
**Sexual function (EPIC-26)**				
T0	64.7 (28.4)	60.9 (28.9)	59.1 (28.5)	59.2 (29.2)
T1	29.5 (26.3)	23.5 (22.0)	22.2 (21.0)	26.2 (24.2)
**Comorbidities**				
Heart disease	280 (5.4)	240 (6.1)	24 (2.3)	114 (10.0)
Other cancer	86 (1.7)	138 (3.5)	32 (3.1)	41 (3.6)
Depression	34 (0.7)	23 (0.6)	2 (0.2)	13 (1.1)
Arthritis	31 (0.6)	15 (0.4)	0 (0.0)	4 (0.4)
Hypertension	1,179 (22.6)	898 (22.8)	96 (9.2)	355 (31.0)
PAD^4^	7 (0.1)	14 (0.4)	3 (0.3)	4 (0.4)
Lung disease	129 (2.5)	82 (2.1)	7 (0.7)	29 (2.5)
Diabetes	251 (4.8)	181 (4.6)	33 (3.2)	50 (4.4)
Kidney disease	71 (1.4)	62 (1.6)	7 (0.7)	25 (2.2)
Liver disease	25 (0.5)	16 (0.4)	4 (0.4)	7 (0.6)
Stroke	5 (0.1)	23 (0.6)	3 (0.3)	7 (0.6)
Disease of the nervous system	60 (1.2)	53 (1.4)	2 (0.2)	3 (0.3)

^1^median (interquartile range) for continuous variables except for EPIC-26 scores (mean and (standard deviation)), absolute (relative) frequencies for categorical variables

^2^category explanation for highest school degree (education): Lower = lower secondary school (incl. German Haupt- and Realschulabschluss), higher = higher secondary school (incl. German Abitur or Fachhochschulabitur)

^3^according to ISUP 2014/WHO 2016 guidelines as recommended by the German S3 guideline for prostate cancer; missing values are referred to as “unknown” if any missingness was observed for the variable; ^4^PAD = peripheral artery disease.

### Prediction of incontinence one year after RP

For the development of the prediction model for incontinence, a sample of *n* = 10 215 was used splitted randomly into a training data set of *n* = 8,191 and a test data set of *n* = 2,024. After a 10-fold cross-validation, *λ* = 0.15 showed the best model fit. Coefficients of the final models can be found in Table 2.

**Table 2:** Coefficients of prediction models for incontinence and sexual function.

	over-all		only robotic RP		only open RP		only laparoscopic RP	
	**Incontinence**	**Sexual Function**	**Incontinence**	**Sexual Function**	**Incontinence**	**Sexual Function**	**Incontinence**	**Sexual Function**
**Intercept**	54.423539043	39.1186616007	75.50073	29.66424	72.69649	23.58196	71.6301	22.63957
**Incontinence (T0)**	0.493140742	0.2847984441	0.510356	0.33436972	0.466824	0.252232135	0.324252	0.226482544
**Age**	-0.403520448	-0.4361009800	-0.49856	-0.5947583	-0.26293	-0.20842942	---	-0.143178782
**Insurance**								
Statutory	reference							
Private	4.424059692	3.471571192	5.705097	2.76125163	2.101949	2.651554989	3.079095	2.683381891
None or other	---		---	---	---	---	---	---
**Citizenship**								
German	reference							
Other	-0.3502792861	---	-3.25592	-0.0866634	---	---	---	---
**Education** ^**2**^								
Lower	reference							
Higher	3.195656395	1.3629308295	1.74047	0.6056317	3.172468	0.564504796	2.830594	---
Other	---	---	2.592007	---	---	---	---	-4.24392436
None	-7.758569891	---	-7.28711	3.50628421	---	---	---	12.23087323
**AS (before RP)**	-7.238429644	-0.5485371605	-6.31822	-1.8192874	---	---	---	---
**ADT (before RP)**	1.855429233	---	0.541663	---	---	---	---	-2.036678409
**WW (before RP)**	---	-0.2292981695	2.483842	---	---	---	---	-9.735979347
**PSA level (at diagnosis)**	---	-0.0037723930	---	-0.0019433	---	-0.001204802	---	-0.051363548
**cT (before RP)**								
T1	reference							
T2	-1.420195564	-1.8379196076	-2.52124	-1.5480338	-0.40307	-1.658691276	---	-2.652880622
T3	-2.330596838	-4.2877742418	-4.87742	-6.1267133	---	-2.265279281	---	---
T4	12.754316978	---	6.868332	---	---	---	---	---
**cN (before RP)**								
N0	reference							
N1	-2.753995350	-2.0924122756	-3.0056	---	---	-5.771610937	---	---
**Gleason score (before RP)** ^**3**^								
Grade 1	reference							
Grade 2	0.407205936	---	0.101056	---	---	0.185967	3.22808	2.571013136
Grade 3	-1.275762207	-2.4219548339	-0.51028	-2.2264389	-0.00057	-2.266509669	---	-1.404151832
Grade 4	-2.634335856	-4.8245182006	-1.69672	-4.8636907	---	-3.666115677	---	-2.265231128
Grade 5	-3.333027890	-4.824582006	-3.041	-6.6564085	---	-0.597388165	---	-1.813081513
**Comorbidities**								
Heart disease	-0.109294918	---	-0.70622	---	---	---	---	-0.001797048
Other cancer	---	---	1.982129	---	---	---	---	-4.117603712
Depression	-0.584768999	-2.1297858818	4.746435	---	-0.44272	---	---	---
Arthritis	-4.551616557	-2.6205121632	-5.15055	---	---	---	---	---
Hypertension	-0.906850853	-0.0002147543	1.013882	---	---	-0.460238959	---	---
PAD^4^	---	---	-10.1882	---	---	---	---	3.953617158
Lung disease	0.505922856	---	-2.36259	-2.7358846	---	---	---	---
Diabetes	-1.748079607	-1.0022589470	-2.79679	-0.7670871	---	-0.09756803	---	-5.429171175
Kidney disease	-1.026948214	---	-4.90635	---	---	---	---	---
Liver disease	---	-1.69747621581	2.170289	-5.4466698	---	---	---	---
Stroke	-9.871983192	-0.0477222306	---	---	-0.75829	---	---	-7.177506326
Disease of the nervous system	-0.002179198	---	---	-0.0801654	---	---	---	---
**Surgical approach**			Not applicable reference					
robotic	Reference							
open	-0.697630169	-3.2746188935						
laparoscopic	-1.675854687	-3.2896973454						

Using the test data set, an internal validation for the model was performed with *R*^2^ = 0.12 and *RMSE* = 25.67 (cf. Table 3). Results of the residual analysis (QQ plots, fitted values versus residuals plots) can be found in S2 File. Regarding calibration-in-the-large, the mean predicted incontinence score was 74.0 compared to an observed mean of 73.9.

**Table 3:** Results of internal and external validation for overall models.

	Incontinence model (T1)		Sexual function (T1)	
	Internal validation *n* = 2,024	External validation *n* = 7,866	Internal validation *n* = 2,097	External validation *n* = 8,081
***R*** ^**2**^	0.12	0.10	0.23	0.22
***RMSE***	25.40	26.03	21.44	21.71

### Prediction of sexual function one year after RP

For sexual function, a sample of *n* = 10 439 was available after imputation of missing predictor values for the development of the prediction model (testing data set *n* = 8 342, training data set *n* = 2 097). The best model fit was chosen for a *λ* = 0.19 (for coefficients, cf. Table 2).

Results from the internal validation are the following: with *R*^2^ = 0.24 and *RMSE* = 21.58 (cf. Table 3 and S2 File). For calibration-in-the-large [28], both mean predicted and mean observed sexual function scores were 26.4.

### External validation of both prognostic prediction models

The temporal external validation for both prediction models is based on a data sample from patients with a T1 questionnaire from June 2021 on (*n* = 8 809)–thus with patients treated after those used for model development. For the incontinence model, *n* = 7 866 observations with a T1 incontinence score were available, for the sexual function model *n* = 8 081. Baseline characteristics (including PRO scores and comorbidities) for the data set used for model development (un-split data set) and for the new data set for external validation can be found in S1 File. Both external validations for the overall models showed comparable results to the internal validations in respect to *R*^2^ and *RMSE* (cf. Table 3). Results of residual analysis of the external validations can be found in S2 File for the overall models. Calibration-in-the-large analysis for the external validation data set was excellent (for incontinence: mean predicted score of 73.7 vs. a mean observed score of 73.4; for sexual: mean predicted score of 26.9 vs. a mean observed score of 26.5).

### Risk of pad use and erectile dysfunction one year after RP

For a more clinically meaningful interpretation of the prediction results, risks of two specific impairments (pad use and erectile dysfunction) were calculated grouped by the corresponding score’s decile. For pad use, the greatest decrease in risk could be observed for the shift between the score categories 50–59 (risk: 95%) and 60–69 (risk: 62.0%). For erectile dysfunction, respectively, the greatest risk decrease was between the categories 30–39 (risk: 57.4%) and 40–49 (risk: 18.7%). Table 4 shows the risks grouped by scores’ deciles.

**Table 4:** Risk of pad use and erectile function one year after RP grouped by corresponding EPIC-26 scores.

Domain score decile^1^	Risk for pad use one year after RP (n patients)	Risk for erectile dysfunction one year after RP (n patients)
0–9	100% (585)	100% (5262)
10–19	100% (363)	97.7% (4093)
20–29	100% (626)	85.6% (1713)
30–39	98.7% (1048)	57.4% (847)
40–49	96.7% (947)	18.7% (237)
50–59	95.0% (1728)	9.9% (92)
60–69	62.0% (694)	2.3% (17)
70–79	50.0% (998)	2.8% (14)
80–89	18.9% (332)	0.7% (3)
90–100	3.5% (234)	0% (0)

^1^For pad use, the corresponding EPIC-26 domain is incontinene, for erectile dysfunction sexual function; both measured one year after RP.

## Discussion

The aim of this study was firstly to develop and validate prognostic models for incontinence and sexual function one year after RP in patients with localised or locally advanced PC and to additionally present prognostic models for different surgical approaches. The two final overall models show adequate internal validation measures; external validation results were comparable and supported the final model fits. The predictive models—if and when used in routine clinical care at prostate cancer centres—are intended to be used to inform patients and clinicians before treatment decisions are made.

Risks for the two of the most important and influential impairments were calculated for deciles of EPIC-26 outcomes additionally [12]. The final models can freely be assessed by clinicians and their patients via an online application (https://nora-tabea.shinyapps.io/EPIC-26-Prediction/) based on the results of this paper.

It is known that although there exist prediction tools, those are rarely used in everyday PC care: clinicians often lack a trust in tools that do not include predictors which are thought to be clinically meaningful–such as comorbidities [30]–or do not include information about clinically relevance [31]. Hence, predictors also rated by clinicians as important–together with already known predictors–were chosen for this study. By applying lasso regression, the most predictive characteristics could be identified for the final models–which serves the scantiness of the final models (being a goal for prognostic prediction tools that should be used in every day clinical care).

**Fig 2:**
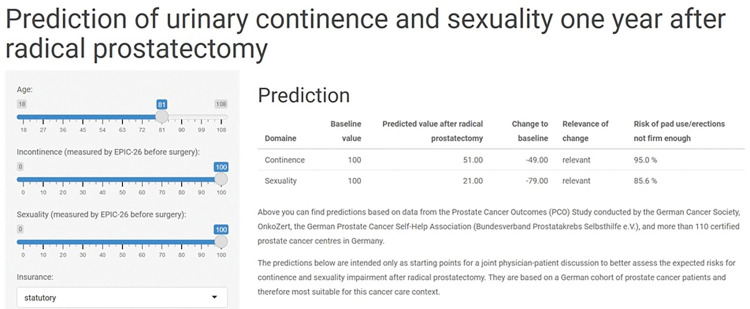
Screenshot of the web-application illustrating how the prediction models could be implemented.

Moreover, by combining predictions with a more detailed information about risks for specific impairments, the proposed prediction tool gets clinically relevant, informative and ready-to-use for urological practice (cf. Fig 2 for screenshot of the web-application).

The presented analysis has several strengths and limitations. First, the presented prediction models were built on very large sample sizes. The analysis includes both an internal and temporal external validation, providing potential future users (clinicians, patients) with two ready-to-implement models freely accessible online for every clinician or interested reader in a web application including information about clinical relevance (cf. link above). In this way, the results presented here can be applied directly in oncological care, e.g. in joint physician-patient discussions [32]. For future initiatives, however, the inclusion of longer timeframes (such as prediction of functional impairments after 24 months) would be desirable to strengthen the possible care implications even more (e.g. Hoffman et al. could show, that especially sexual impairment continues to improve after more than one year [33]). Moreover, the inclusion of other treatment options (such as radiation or active surveillance) could improve the prediction tool even further. However, following Scholl et al. model of patient-centredness [34], the proposed prediction tool is understood as an enabler of a better “patient information” for patients with planned RP and not as a decision tool discriminating between different treatment options.

Furthermore, with the statistical method used for model development (lasso regression) a state-of-the-art approach was applied to ensure a rigorous predictor selection and avoid over-fitting. Especially the latter is an important feature since it increases the chances that the final models do work in similar but different populations.

Lastly, this analysis includes a temporal external validation. Although the importance and need for external validation cannot be overemphasised [29], it should be noted for the present analysis that the data sets used for validation were very similar since the same data collecting infrastructure was used. Thus, results may not be too generalisable to populations outside Germany or not treated by a comparable health care provider (i.e., a specialised centre for on-ward PC care).

Although predictors were selected from a set of covariates that are clinically useful, the fact that these covariates have been selected for the final prediction models does not indicate any causal relationship with the outcomes (thus, coefficients are not further interpreted and only used for prediction purposes). Further research using a causal framework is needed to understand whether specific interventions result in more beneficial outcomes for patients [35]. Some known predictors like BMI were not available. In-depth information about surgery (i.e. experience of the surgeons) as well as predictors that need higher-level clinical information (i. e. length of membranous urethra) were not included since the tool should be accessible for patients, as well. If–in future care settings–for instance more and higher quality imaging data from diagnosis is available for many patients, other relevant predictors of functional outcomes that could already show their predictive properties within smaller studies [36] could be included, as well. Other known predictors of functional outcomes after RP directly connected to surgery, were not included on purpose: The decision of specific surgical techniques (i.e. nerve-sparing techniques) applied is ultimately often only made during surgery and not beforehand. Thus, for a prediction tool that is supposed to be used before treatment initiation, those information is not available and cannot be used for prediction purposes (although it has an impact on the outcome [37]). Not including those possible predictors may be one reason for the unexplained variance of our prediction models.

To conclude, important functional outcomes after RP for localised or locally advanced prostate cancer can be predicted before treatment initiation using the proposed models and the ready-to-use web-application. The proposed models are based on very large sample sizes ensuring a robust prediction. Combined with the risks of specific prostate specific impairments for predicted outcomes, the targeted use of the decision-support for improved, informed shared decision-making by patients and their treating physicians is enabled.

## Supporting information

S1 File Results for models stratified by surgical approach. (DOCX)

S2 File Residual analysis. (DOCX)

S3 File Missing values analysis. (DOCX)

S4 File Variable importance of over-all models. (DOCX)

S5 File TRIPOD table. (DOCX)
