# Supplementary material for: Prediction models of incontinence and sexual function one year after radical prostatectomy based on data from 20 164 prostate cancer patients
Source: PLoS One. 2023 Dec 1;18(12):e0295179. doi: 10.1371/journal.pone.0295179 (PMC10691723; doi:10.1371/journal.pone.0295179)
Supplement: S1 File — (DOCX) [file pone.0295179.s001.docx]

# Supplementary Material S1: Results for models stratified by surgical approach

|  | **Incontinence model (T1) open** | | **Sexual function (T1) open** | |
| --- | --- | --- | --- | --- |
|  | **Internal validation**  $\boldsymbol{n=}$ **3560** | **External validation**  $\boldsymbol{n=}$ **2561** | **Internal validation**  $\boldsymbol{n=}$ **3644** | **External validation** $\boldsymbol{n= 2632}$ |
| $\boldsymbol{R}^{\boldsymbol{2}}$ | 0.09 | 0.10 | 0.18 | 0.17 |
| $\boldsymbol{RMSE}$ | 26.20 | 26.75 | 19.83 | 20.05 |

|  | **Incontinence model (T1) robotic** | | **Sexual function (T1) robotic** | |
| --- | --- | --- | --- | --- |
|  | **Internal validation**  $\boldsymbol{n=}\boldsymbol{4715}$ | **External validation**  $\boldsymbol{n=}$ **3947** | **Internal validation**  $\boldsymbol{n=}$ **4861** | **External validation** $\boldsymbol{n=}$ **4043** |
| $\boldsymbol{R}^{\boldsymbol{2}}$ | 0.13 | 0.12 | 0.24 | 0.24 |
| $\boldsymbol{RMSE}$ | 24.69 | 25.27 | 22.83 | 22.81 |

|  | **Incontinence model (T1) lap** | | **Sexual function (T1) lap** | |
| --- | --- | --- | --- | --- |
|  | **Internal validation**  $\boldsymbol{n=919}$ | **External validation**  $\boldsymbol{n=}$ **613** | **Internal validation**  $\boldsymbol{n= 946}$ | **External validation** $\boldsymbol{n=}$ **641** |
| $\boldsymbol{R}^{\boldsymbol{2}}$ | 0.03 | 0.07 | 0.16 | 0.16 |
| $\boldsymbol{RMSE}$ | 25.13 | 26.63 | 19.30 | 19.29 |
