# Supplementary material for: Prediction models of incontinence and sexual function one year after radical prostatectomy based on data from 20 164 prostate cancer patients
Source: PLoS One. 2023 Dec 1;18(12):e0295179. doi: 10.1371/journal.pone.0295179 (PMC10691723; doi:10.1371/journal.pone.0295179)
Supplement: S2 File — (DOCX) [file pone.0295179.s002.docx]

# Supplementary Material S2: Residual analysis


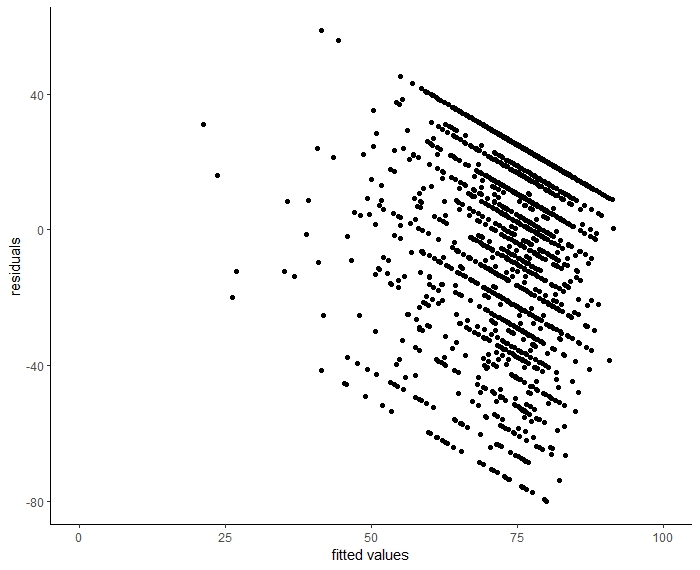


Figure 1: Residual analysis of internal validation of the final model for incontinence (T1) for n = 2,024; fitted values from the final model vs. residuals


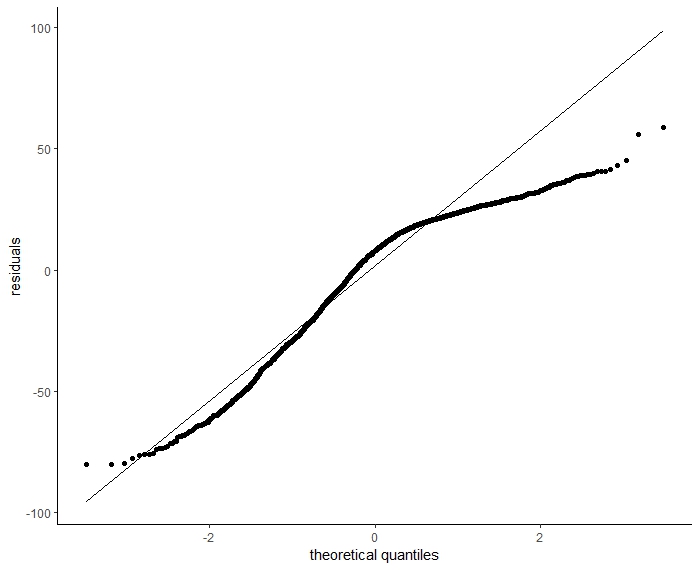


Figure 2: Residual analysis of internal validation of the final model for incontinence (T1) for n = 2,024; QQ plot showing distribution of residuals


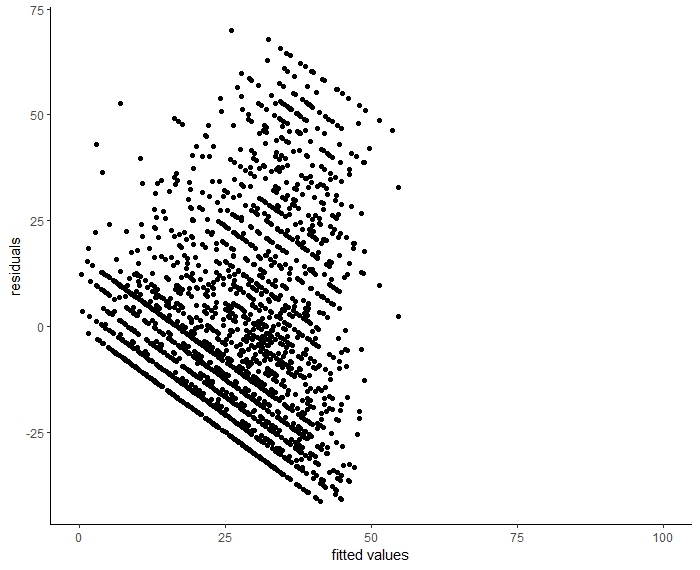


Figure 3: Residual analysis of internal validation of the final model for sexual function (T1) for n = 2,097; fitted values from the final model vs. residuals


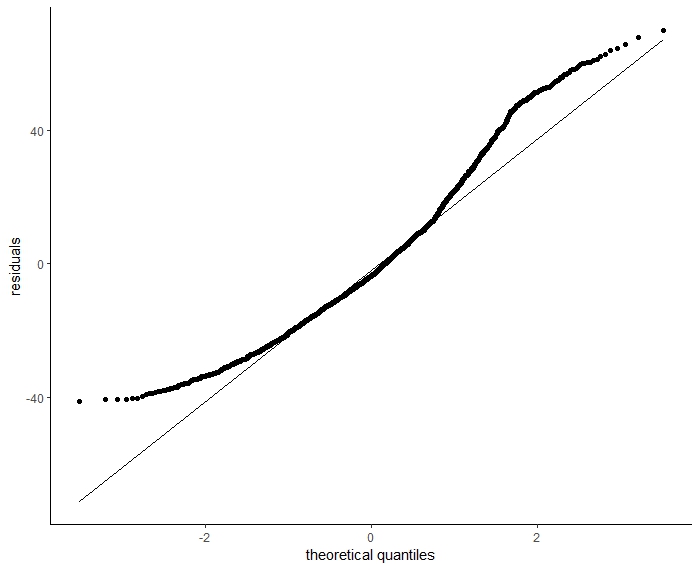


Figure 4: Residual analysis of internal validation of the final model for sexual function (T1) for n = 2,097; QQ plot showing distribution of residuals


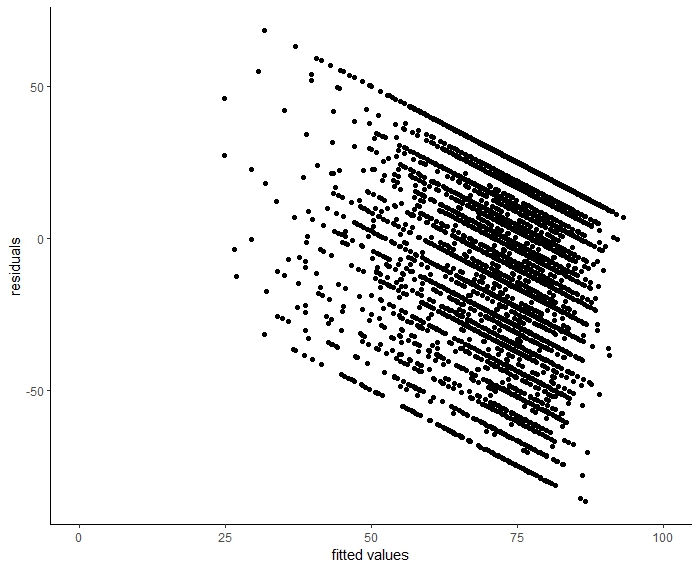


Figure 5: Residual analysis of external validation of the final model for incontinence (T1) for n = 7,866; fitted values from the final model vs. residuals


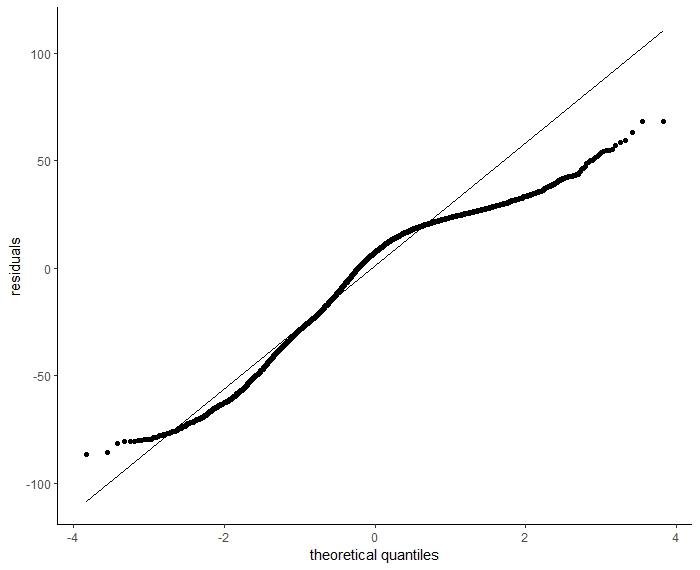


Figure 6: Residual analysis of external validation of the final model for incontinence (T1) for n = 7,866; QQ plot showing distribution of residuals


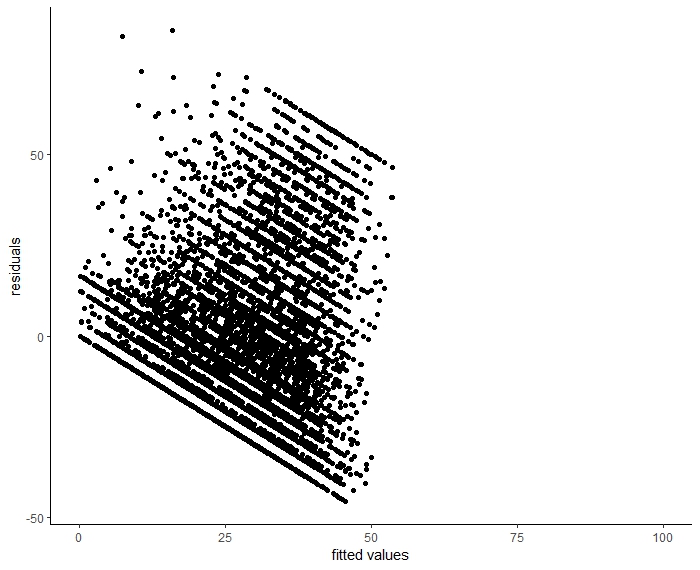


Figure 7: Residual analysis of external validation of the final model for sexual function (T1) for n = 8,081; fitted values from the final model vs. residuals


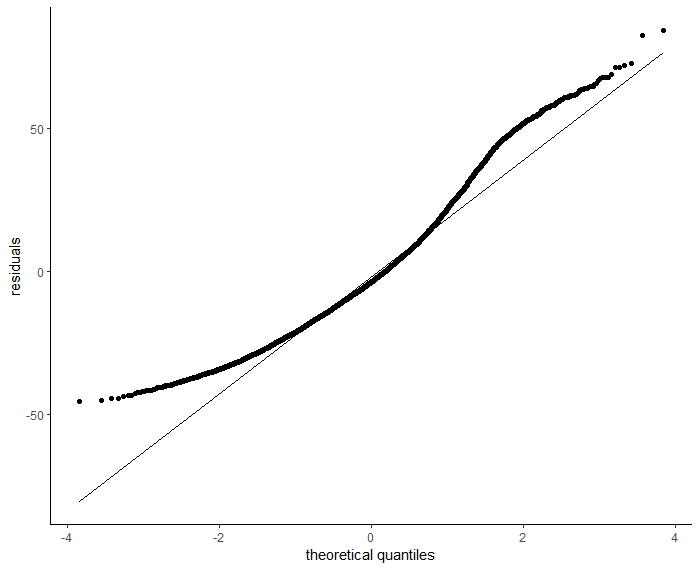


Figure 8: Residual analysis of external validation of the final model for sexual function (T1) for n = 8,081; QQ plot showing distribution of residuals
