# Supplementary material for: Prediction models of incontinence and sexual function one year after radical prostatectomy based on data from 20 164 prostate cancer patients
Source: PLoS One. 2023 Dec 1;18(12):e0295179. doi: 10.1371/journal.pone.0295179 (PMC10691723; doi:10.1371/journal.pone.0295179)
Supplement: S3 File — (DOCX) [file pone.0295179.s003.docx]

# Supplementary Material S3: Missing values analysis

For this analysis, data from the PCO study for $n = 11,355$ patients was used. The PCO study combines existing certification data with patient- and centre-documented survey data. Certification of the DKG requires required documentation of many process- and structural data on an individual patient level. Thus, the following variables do not have any missing values: age, cT, cN, Gleason score, AS, WW or ADT before surgery, and surgical approach. Additionally, due to the documentation of comorbidities for the PCO study (centre-documented with only the options yes or no), no missing information for comorbidities is available.

Consequently, absolute frequencies and percentages of missing values for the following variables can be found in table 1 and figure 1 of this supplementary material: insurance, education, citizenship, PSA (at diagnosis), and EPIC-26 scores (T0 and T1).

| Variable | Missing values (%^1^) |
| --- | --- |
| Insurance | 320 (2.82) |
| Education | 329 (2.90) |
| Citizenship | 321 (2.81) |
| PSA level (at diagnosis) | 1 (< 0.01) |
| Incontinence (T0) | 537 (4.73) |
| Incontinence (T1) | 285 (2.51) |
| Sexual function (T0) | 359 (3.16) |
| Sexual function (T1) | 175 (1.54) |

Table 1: Absolute and relative frequencies of missing values; ^1^for n = 11,355 patients

Figure 1 moreover displays a graphical illustration of patterns of missingness (in total: 53 different combinations). The diagrams were generated with the help of the aggr function of the R package VIM.


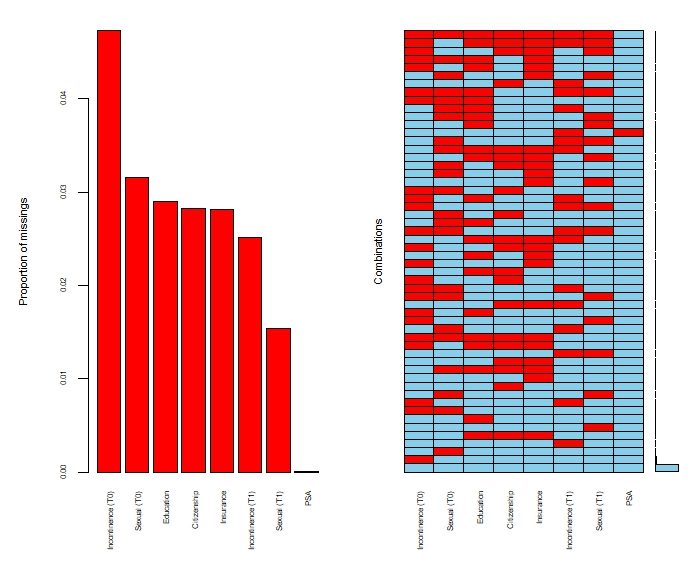


Figure 1: Proportions of missing values and pattern plot for combinations of missing values for the data set for model development (n = 11,355)
