# Supplementary material for: Prediction models of incontinence and sexual function one year after radical prostatectomy based on data from 20 164 prostate cancer patients
Source: PLoS One. 2023 Dec 1;18(12):e0295179. doi: 10.1371/journal.pone.0295179 (PMC10691723; doi:10.1371/journal.pone.0295179)
Supplement: S4 File — (DOCX) [file pone.0295179.s004.docx]

# Supplementary Material S4: Variable importance of over-all models

To present the importance of the single predictors for the proposed models, the variable importance of the predictors is shown below by using the absolute values of the shrinked, centred and scaled coefficients and scaling them (between 0 and 100 %) using the *varImp* function from the *caret* R package (for glmnet objects). For this purpose, the most influential predictor is used as the reference category and the remaining predictors are than scaled according to this predictor.


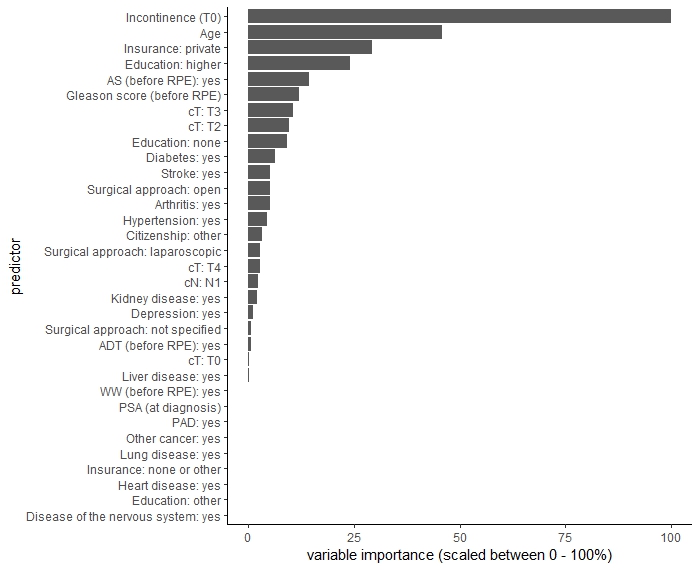


Figure 1: Variable importance for the final lasso model for incontinence (T1); variable importance is based on the absolute value of the shrinked coefficients; predictors without a bar have coefficients shrinked towards 0; PAD = peripheral artery disease


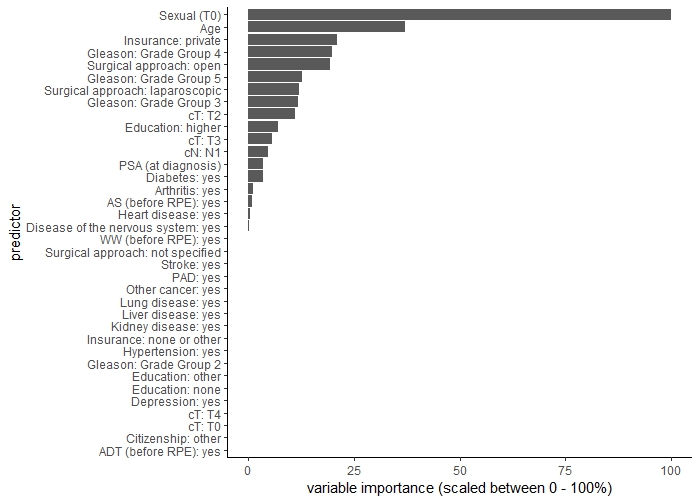


Figure 2: Variable importance for the final lasso model for sexual function (T1); variable importance is based on the absolute value of the shrinked coefficients; predictors without a bar have coefficients shrinked towards or very close to 0; PAD = peripheral artery disease
